# A domain-based approach to predict protein-protein interactions

**DOI:** 10.1186/1471-2105-8-199

**Published:** 2007-06-13

**Authors:** Mudita Singhal, Haluk Resat

**Affiliations:** 1 Computational Biology and Bioinformatics Group, Pacific Northwest National Laboratory, P.O. Box 999, MS: K7-90, Richland, WA, 99352 USA

## Abstract

**Background:**

Knowing which proteins exist in a certain organism or cell type and how these proteins interact with each other are necessary for the understanding of biological processes at the whole cell level. The determination of the protein-protein interaction (PPI) networks has been the subject of extensive research. Despite the development of reasonably successful methods, serious technical difficulties still exist. In this paper we present DomainGA, a quantitative computational approach that uses the information about the domain-domain interactions to predict the interactions between proteins.

**Results:**

DomainGA is a multi-parameter optimization method in which the available PPI information is used to derive a quantitative scoring scheme for the domain-domain pairs. Obtained domain interaction scores are then used to predict whether a pair of proteins interacts. Using the yeast PPI data and a series of tests, we show the robustness and insensitivity of the DomainGA method to the selection of the parameter sets, score ranges, and detection rules. Our DomainGA method achieves very high explanation ratios for the positive and negative PPIs in yeast. Based on our cross-verification tests on human PPIs, comparison of the optimized scores with the structurally observed domain interactions obtained from the iPFAM database, and sensitivity and specificity analysis; we conclude that our DomainGA method shows great promise to be applicable across multiple organisms.

**Conclusion:**

We envision the DomainGA as a first step of a multiple tier approach to constructing organism specific PPIs. As it is based on fundamental structural information, the DomainGA approach can be used to create potential PPIs and the accuracy of the constructed interaction template can be further improved using complementary methods. Explanation ratios obtained in the reported test case studies clearly show that the false prediction rates of the template networks constructed using the DomainGA scores are reasonably low, and the erroneous predictions can be filtered further using supplementary approaches such as those based on literature search or other prediction methods.

## Background

Understanding biological processes requires knowing not only which proteins exist in a certain organism or cell type but also how these proteins interact with each other. However, determining the protein-protein interaction (PPI) networks is a daunting task and has been the subject of extensive research. Many computational and experimental techniques have been developed to observe or predict the PPI networks in biological systems [1-5]. Despite the development of reasonably successful methods, serious technical difficulties still exist. Arguably, the small overlap between the two major high-throughput experimental approaches, the two-hybrid systems [1,6] and co-immunoprecipitation of protein complexes [7,8], best reflects these difficulties [2,9]. Weak and non-specific spurious interactions in both classes of experiments cause additional significant technical challenges [3,7], and it has been estimated that less than half of the interactions observed in high-throughput experiments are true predictions [10,11]. On the computational side, because of the sheer size difference between negative (non-interacting pair) and positive (true interaction) PPIs, even a very low false-positive prediction rate can result in a situation where most of the predicted interactions are incorrect [12].

To overcome such problems, the use of various intersecting data types [4,5,9,11-17] as well as the use of interaction data for multiple organisms tied via homology-based approaches [5,18-21] has been proposed to improve the PPI networks. The information content hidden in the structural properties of proteins has also been used to construct and improve the PPI networks [18,22]. In this report, we expand on this latter feature and advocate a quantitative approach that depends on structural domains of proteins as a fundamental filtering step in inferring biological PPI networks. The underlying premise in our approach is that proteins interact with each other through their smaller substructures (i.e., domains), which have the biophysical properties that are instrumental in protein-protein complex formations [23]. The validity of this assumption stems from the fact that evolutionarily conserved polypeptide domains can be thought of as structural building blocks that define and regulate the functionality of the proteins. Such ideas also form the foundation of the databases, such as the Pfam database [24], allocated to the characterization of protein domains.

Simply put, in the domain-based structural quantification approach, the knowledge about the strength of the interaction between domain d_i _in protein X and domain d_j _in protein Y is used to predict whether proteins X and Y interact. Corollary to this would be that for a given list of PPIs, can the possible interactions (and their strengths) between domain pairs be determined? This idea has been researched recently [58,59] and was behind the development of the InterDom database [25,26], and it forms the starting point of our study presented here. We first develop a scoring scheme for the interactions between the functional domains of the proteins and then use it to predict the strength of interaction between protein pairs.

Several methods have been proposed for correlating domain pairs based on their frequency of occurrence in interacting protein pairs [25,27,28] or by their probability of interaction [29]. As shown by Wojcik et al., domain-domain interactions are good indicators of possible protein interactions, and they can be used to predict the protein interactions more accurately than approaches that use comparison of full-length protein sequences [30]. Maximum Likelihood Estimation based methods to infer domain interactions have been developed and used to predict the interactions between yeast proteins [28,29]. Domain interactions have also been described with an attraction-repulsion model [31]. Ng et al. devised an integrative approach to computationally infer protein domain interactions and showed that the use of heterogeneous data sources improved protein interaction detection sensitivity [25]. Recently, Riley et al. have presented the domain pair exclusion analysis method for inferring domain interactions from multiple organisms using the Database of Interacting Proteins (DIP) [32]. Other efforts have further extended this approach [17,33,34]. Guimarães et al. used a parsimony approach combined with linear programming formulation to derive the statistical scores for the domain interactions [33]. They also discuss crucial aspects of evaluation of the predictions as well as ways to group the interaction data into categories based on their difficulty of predictions [33]. Lee et al. combines the data from multiple organisms in their integrated approach [17], and Wuchty utilizes the information about the topology of the network in predicting the protein interactions [34].

Effort behind the development of InterDom [26], a database of putative domain-domain interactions, differs from the above mentioned statistical methods by attempting to directly quantify the strength of the domain-domain interactions. In the InterDom database, the domain-domain interactions are derived by combining data from multiple sources: domain fusions, protein interactions and complexes, and scientific literature. A probability-based scoring scheme is used to assign higher confidence to domain interactions that are derived independently by multiple methods from different data sources [25,26]. Although it is a novel effort, our analysis results show that the InterDom scores need significant improvements before they can be used for predictive purposes with reasonable precision.

In this study we present the DomainGA method, a Genetic Algorithm type machine-learning approach that quantifies the protein domain-domain interactions. Our algorithm generates a set of domain-domain interaction scores, which are then used to classify the interactions into three categories: high, low, and fuzzy. As in any machine-learning technique, our approach requires good-quality training data. Because a large quantity of publicly available data exists, *Saccharomyces cerevisiae *(yeast) is arguably the best model organism for testing the new algorithms. We therefore benchmark our algorithm using the PPI data available for *S. cerevisiae*.

In the following sections, we first demonstrate why a new domain-domain scoring scale is needed. We then present our results that show the robustness, accuracy, and success of our DomainGA algorithm. Details of the DomainGA algorithm and how it was numerically implemented can be found in the Methods section.

## Results and Discussion

### A. Evaluation of InterDom scores

**Figure 1:**
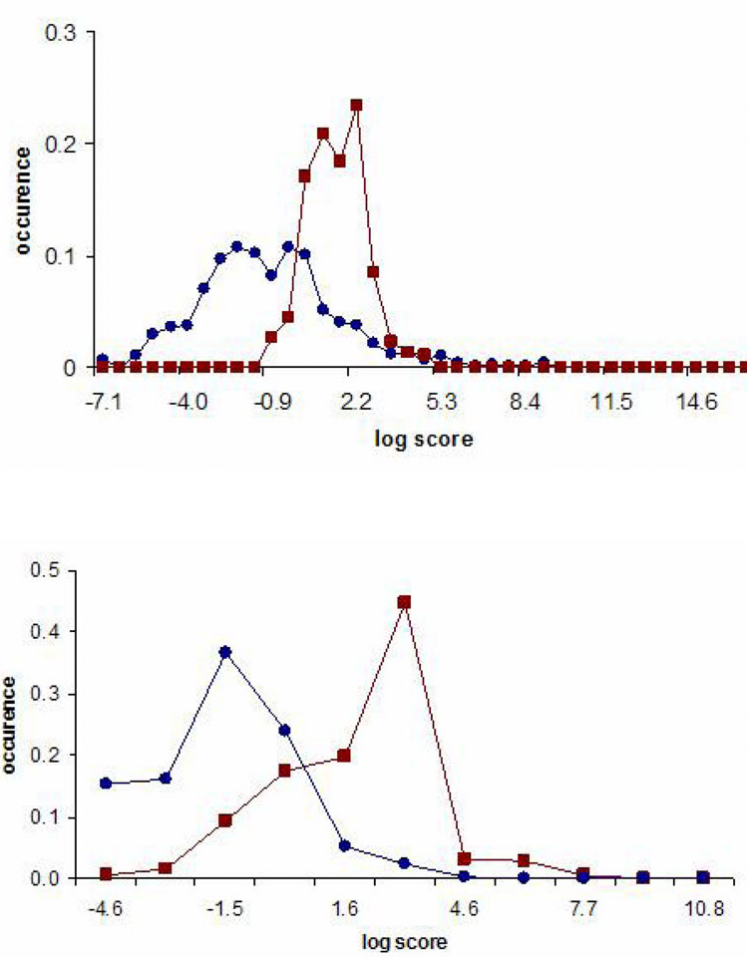
Comparison of the strengths of the Munich Information Center for Protein Sequences (MIPS) positive (red line with squares) and negative (blue line with circles) protein-protein interactions computed using the InterDom domain-domain interaction scores. The interactions with a score of zero are not reported. The histogram curves were calculated by binning the logarithm of the protein-protein interaction scores that were computed using the maximum-score detection rule. Vertical axis shows the percentage of the PPIs with interaction scores that are within the strength interval of a particular bin. Top: Yeast PPI; Bottom: Human PPI.

The InterDom database contains a set of scores for domain-domain pair interactions [26]. This set can be used to evaluate the scores in terms of their predictive power of the protein-protein interactions. If the domain interaction scores have good discriminatory power, predicted protein-protein interaction scores for the positive and negative PPI lists should be different – at least qualitatively. However, as Figure 1 shows, score distributions for the negative and positive lists for the human and yeast PPIs have considerable overlap. We note that this analysis overlooks certain factors that are also determinants of domain-domain, and therefore protein-protein, interactions. Therefore, the lack of a clear separation between the scores for the positive and negative PPI predictions may not be entirely due to the InterDom scores. Subtle differences in actual domain structures such as the ones due to amino acid composition, environmental factors, and whether the placement of the domain is in an accessible portion of the protein would be a few of such factors. For these reasons, not a complete but only a reasonable separation between the curves is to be expected in Figure 1. However, the observed large overlap clearly indicates that there is room for improving the InterDom domain-domain interaction scores.

### B. Dependence on the optimization procedure

Our Domain GA approach is a multi-parameter optimization method in which the extreme value of a fitness (score) function is searched. Adapting the domain-domain interaction scores to predict PPIs requires the development of a criterion for deciding what domain-domain score corresponds to a PPI. For this, we first form a list of all possible domain-domain interactions between two proteins; that is, all possible combinations between domain pairs. We then take the largest or the total of the domain-domain interaction scores to represent the ***strength ***of the interaction between the two proteins. If the determined strength is larger than a predetermined cutoff value, we classify the protein pair as interacting and as non-interacting otherwise. We use the term *strength *in an unconventional manner. In our case, it is a score that represents the likelihood of interaction between two domains. The likelihood however has a bounded range and it is discretized for practical implementation. Depending on the representation, our score can be interpreted as a normalized and scaled biochemical binding affinity or the thermodynamic Boltzmann factor, or as the statistical interaction probability. In the future versions of the algorithm, we plan to use a continuous and unbound score range, which would make these correspondences more obvious.

Unless indicated otherwise, in all reported cases a ***maximum-score detection rule ***was used during the optimization step. The maximum-value detection rule refers to the optimization score where the predicted category assigned to each PPI was based on the maximum score among all of the pairwise domain pairs for a given protein couple. In other words, the maximum value of any possible combination of domain pairs that can be formed between the involved proteins is used to decide whether two proteins interact.

Because of the small size of the available training data, it is infeasible to include all possible domain pairs. We therefore significantly reduce the parameter set to avoid over-fitting the parameters during the optimization step. Using only a very small fraction of the whole parameter set raises the question of how dependent the derived values are on the size of the defined parameter set. A related concern is how representative the small set can be in terms of explaining the observations that are used as the training data. To address these issues we have performed test case studies to show that our selection procedure is reasonable.

#### Invariance with respect to the parameter score range

**Figure 2:**
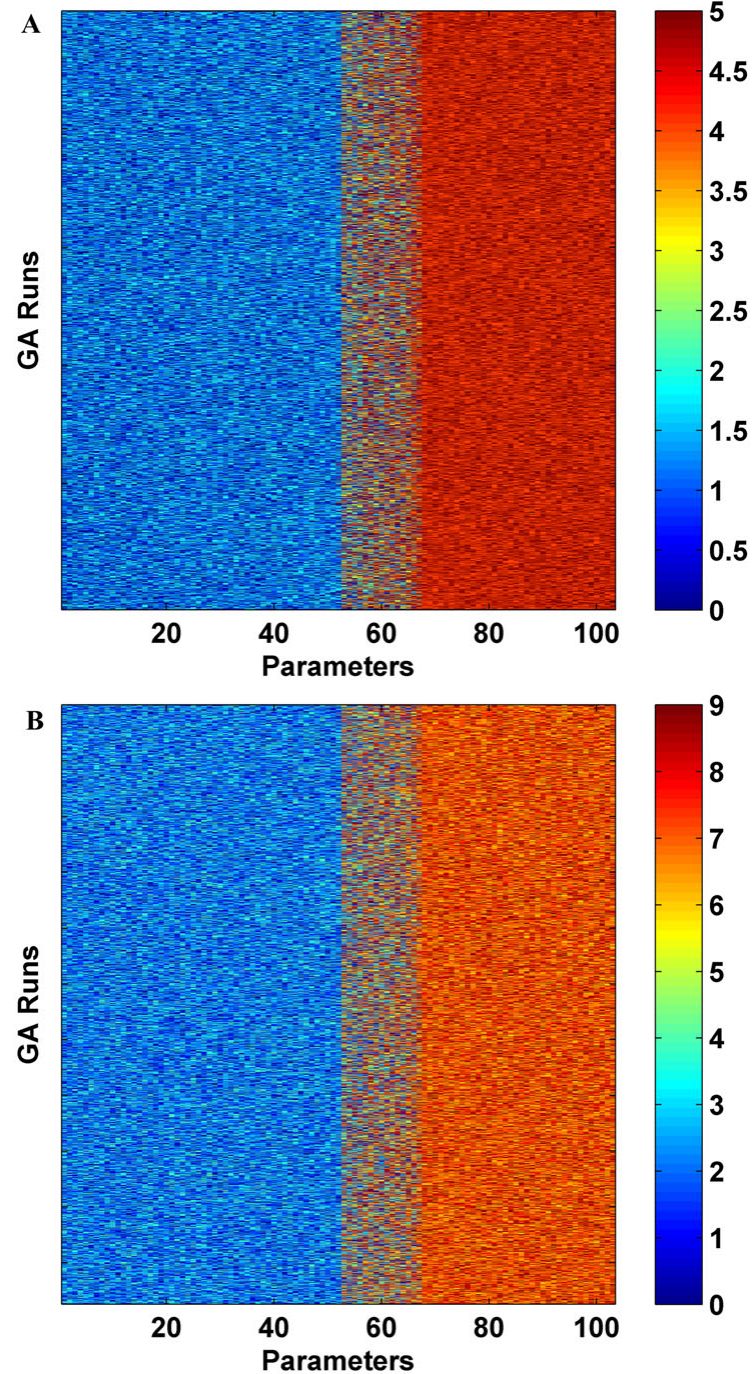
Comparison of the scores of the common 103 parameters that were optimized using different ranges for the scores with the inclusive set. Employed range was: (A) [0–5] and (B) [0–9]. In the figures, the vertical axis represents a particular GA run and the horizontal axis shows the optimization parameters, which are rank ordered according to their mean strength values. Each column shows the score of a particular parameter obtained in different GA runs. A consistent color through a column indicates that the optimized value of corresponding parameter is almost the same in all the GA runs. Each plot reports the optimized score set values for more than 2,000 GA runs. Intense blue and red colors respectively represent the non-interacting and interacting domain-domain pairs. The Yeast MIPS dataset compiled by Jansen et al. was used.

In our DomainGA, parameter values (i.e., the strength of each domain pair interaction) are optimized to maximize agreement with the training PPI list used. Although the use of a continuous range for the scores is possible, reverting to a discrete scale is more convenient for searching the parameter space. Therefore, in the current implementation of DomainGA, we allow the parameters to have integer values between 0 and T, where the upper bound determines the coarseness of the discretization. Figure 2 compares the results for the smallest data set when the maximum score value T was chosen as 5 and 9. The cutoff value to decide whether possible domain-domain interactions result in a PPI was chosen as the mid-values 3 and 5 for the T = 5 and 9 cases, respectively. Choosing the mid-values as the cutoff was totally arbitrary. In Fig. 2, the parameter scores are reported using a color scheme and the order of the parameters is the same in both parts. Each row in Fig. 2 shows the values of the parameter set (i.e., the domain interaction scores) optimized in a particular GA run. Each column shows the optimized value of a particular parameter across different GA runs. A uniform color through a column means that the corresponding parameter's score remain consistent across many different GA runs. Dominant red and blue colors represent interacting and non-interacting domain pairs, respectively, and other color shades correspond to intermediate parameter scores. We define the parameters with intermediate scores or whose values fluctuate between high and low scores across the different GA runs as fuzzy (or indefinite) parameters. It is clear from Fig. 2 that the scale choice does not make a noticeable difference. A correlation analysis of the optimized parameter values computed as the mean of the GA runs indicates an almost perfect match with an R-square value of 0.9996 between the T = 5 and 9 cases.

#### Invariance with respect to the number of parameters

**Figure 3:**
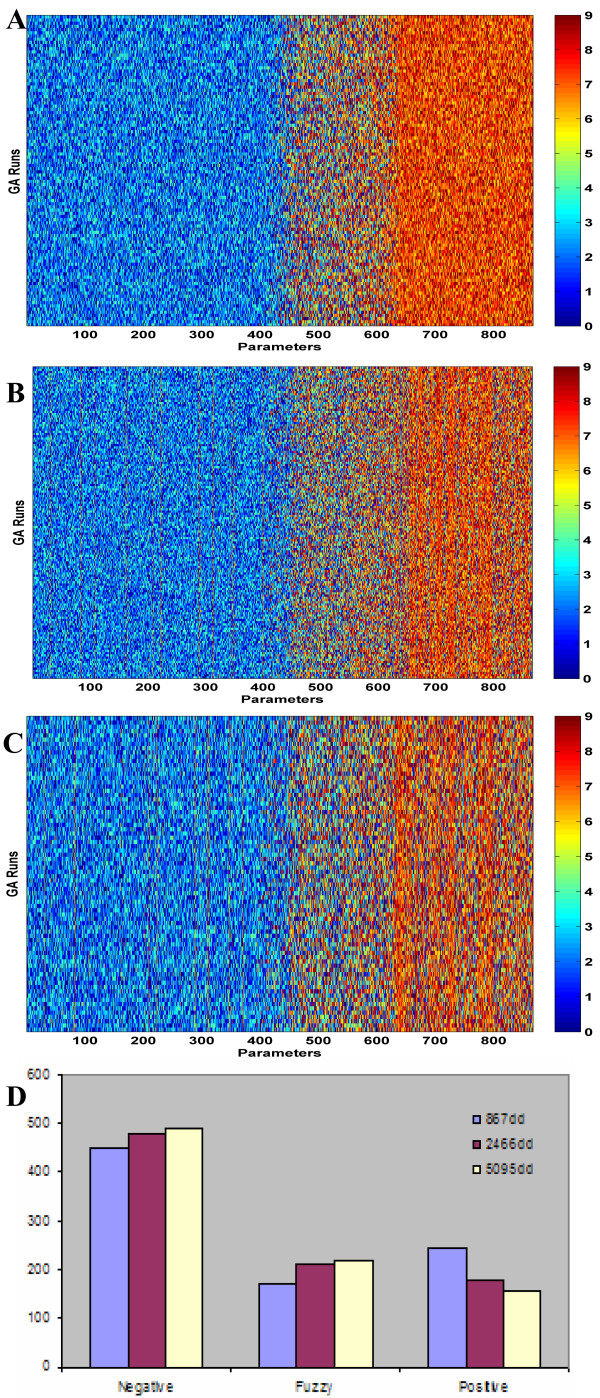
Score comparison between the optimization studies with different number of parameters. Similar to Figure 2, parts (A-C) report the scores of the 867 parameters that were common in all three cases. Inclusive set optimizations with: (A) 867; (B) 2466; and (C) 5095 parameters. Part (D) reports and compares the classification of the optimized scores according to their interaction profiles.

In an optimization study, an added concern is the dependence on the size of the parameter set. To address this issue, we have created datasets with different number of parameters, Table 1. As discussed in the Methods section, dataset with 867 parameters was selected based on single- and multiple-occurrence statistics of the domain pairs in the training set. The size of this dataset was further increased to 2466 and then to 5095 by adding more parameters to the list (Table 1). We note that the parameters of the 867-parameter set are a subset of the larger parameter sets. Inclusion of the same parameters in several datasets allowed us to test numerically whether the optimized values of the parameters depend on the size of the set used. Figure 3 reports the optimized values for the 867 parameters that are common in all sets. Comparison of the results shows that the assignments of a small fraction (~15%) of the parameters change between the high, low, or fuzzy categories. Therefore, vast majority of the domain-domain pair interaction scores do not depend on the included number of optimization parameters. The most noticeable pattern between the results for the cases is that, as the number of optimized parameters is increased, scores of some of the parameters shift from the positively determined to the fuzzy (indeterminate) category, Fig. 3D. The differences however do not alter the explanation ratios of the training datasets, Table 2.

**Table 1:** Details of the yeast MIPS datasets used in optimization studies *

**No of Parameters/Data Set **^a^		**PPI**	**Retained Interactions**
103	Inclusive	Positive	342
		
		Negative	14,402

867	Inclusive	Positive	1,882
		
		Negative	79,413

344 ^a^	Closed	Positive	435
		
		Negative	3,139

2466	Inclusive	Positive	2,308
		
		Negative	162,115

1216 ^a^	Closed	Positive	734
		
		Negative	13,146

5095	Inclusive	Positive	2,666
		
		Negative	243,866

3060 ^a^	Closed	Positive	1,448
		
		Negative	25,651

* Starting yeast dataset was obtained from Munich Information Center for Protein Sequences (MIPS) site [35] and it contained 8250 positive and ~2 million negative protein-protein interactions [12]. Retained interactions column report the number of entries for the sets after the original dataset is filtered according to the domain pairs included as optimization parameters. Further details can be found in the Methods section. ^a ^These are the closed set versions of the 867, 2466, and 5095 parameter inclusive sets. As explained in the Methods section, during filtering to obtain the closed PPI sets, occurrence of some of the domain pairs are nullified and these parameters cannot be truly optimized during the GA runs. So these closed sets are a subset of their corresponding inclusive sets.

**Table 2:** Explanation ratios of the MIPS yeast datasets

**No of Parameters**	**Training set**	**PPI**	**Explanation Ratio (%) **^a^	**Accuracy**	**Precision**
867	Inclusive	Positive	95.6	96.4	38.9
				
		Negative	96.1		

344	Closed	Positive	99.3	98.7	90.6
				
		Negative	98.7		

2466	Inclusive	Positive	96.2	95.7	24.3
				
		Negative	95.7		

1216	Closed	Positive	99.0	96.6	60.8
				
		Negative	96.4		

5095	Inclusive	Positive	97.3	95.6	19.3
				
		Negative	95.6		

3060	Closed	Positive	99.3	95.9	56.5
				
		Negative	95.6		

Deng et al. with	Inclusive	Positive	95–98	93–95	24–30
				
867 pmts ^b^		Negative	93–95		
	
	Closed	Positive	91–93	89–90	54–55
				
		Negative	89–90		

Random with	Inclusive	Positive	61.0	36.0	2.2
				
867 pmts		Negative	35.4		

^a ^Explanation ratio is the ratio of successful predictions to the overall number of entries in a particular list. We note that the explanation ratios for the positive and negative PPI lists respectively correspond to the sensitivity and specificity with Lin et al. definitions.

^b ^Calculated performance metrics depend on the false positive and negative prediction rates used in the MLE algorithm as well as on the cutoff for positive and negative PPI assignments. Therefore, we report the range of obtained percentages that were obtained when various prediction rates were used in the MLE algorithm. Whole set of results can be found in Supplementary Table 1 [see Additional file [Supplementary-material S1]].

#### Invariance with respect to the detection rule

We test the robustness of the DomainGA method with respect to the detection rule choice by developing the ***total-score detection rule***. As its name implies, in the total-score detection rule, the predicted category assigned to each PPI was based on the total scores of all the domain pairs in that protein pair. In other words, rather than picking the maximum value among the possible combination of domain pairs, the domain-domain interaction scores were summed. In terms of biophysical considerations, the maximum-score detection rule emphasizes the dominant domain-domain interaction, and it implicitly assumes that proteins interact through, at most, one domain at a time, and the domain-domain pair with the highest affinity is the most crucial one. In contrast, the total-score detection rule considers all possibilities by summing over the interaction score, which is analogous to calculating the cumulative thermodynamic free energy of a PPI where every possibility contributes according to its strength.

**Figure 4:**
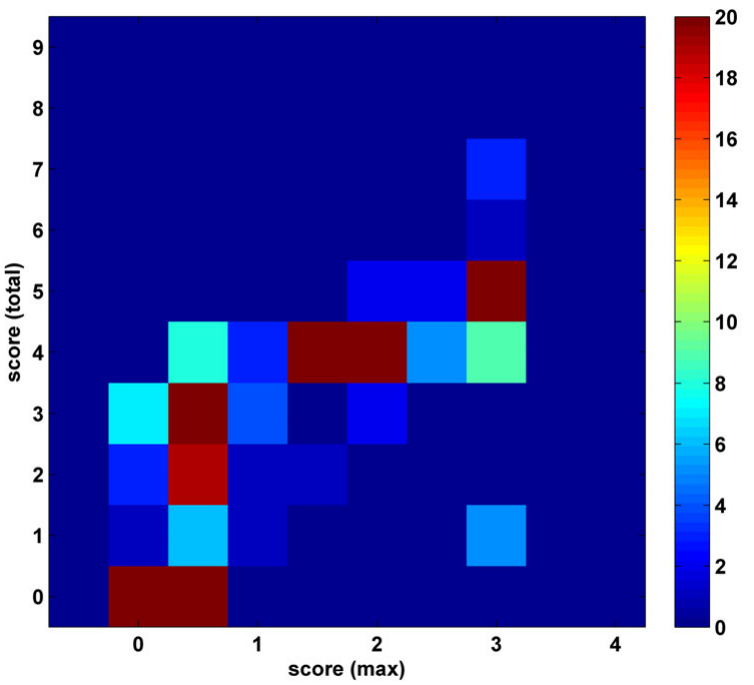
Comparison of the parameter scores optimized using the 344 parameter closed set with maximum (x-axis) and total (y-axis) score detection rules. Reported scores are the averages of the GA runs after the infrequently occurring parameter values are discarded during analysis. Histogram diagram reports the score distribution of the parameters that can be optimized in the simulations. Each (*x,y*) entry in this histogram plot reports the number of parameters that has mean values of *x *and *y *when the maximum- and total-score detection rule was used in the optimization, respectively. The maximum value of the color scale is lowered from 67 to 20 to enhance the contrast between the histogram points. The yeast MIPS dataset compiled by Jansen et al. was used.

Optimizations using both of the detection rules were carried out using the closed 344 parameter set (Table 1). The parameter score range was [0–9] and a cutoff of 5 was used to classify the PPIs into positive or negative interaction categories. Parameter values obtained using the total- and the maximum-score detection rules are compared in Figure 4. As the reported two-dimensional histogram shows, the scores of the domain pairs in these two optimization studies lie close to the diagonal demonstrating the promise that the DomainGA results are rather insensitive to the detection rule. There are only a few parameters that have conflicting optimized values between the two detection rule cases. These appear as a spike at the (max ~7, total ~1) point in the histogram diagram indicating a discrepancy between the parameter sets. We note that the small differences at the low or high parameter scores are unimportant because in the current classification scheme, values are simply grouped into three classes: non-interacting (< 5), fuzzy (~5), and interacting (> 5). Therefore, small variations in the (0:3) or (7:9) ranges are irrelevant to the derived conclusions.

### C. Predictions of the DomainGA method

In the previous sections we have shown the robustness and insensitivity of the DomainGA method to the selection of the parameter sets, score ranges, and detection rules. The details of the Munich Information Center for Protein Sequences (MIPS) datasets [12,35] that were used as the training and testing data in our computations are tabulated in Table 1. We note that the larger sets with 2466 and 5095 parameters contain domain pairs that occur rather infrequently in the protein interaction list that is used as the training data. This leads to having limited amount of information to optimize these parameters. Thus, judging from the domain occurrence counts, we find the inclusive parameter set with 867 elements to be a reasonable compromise between having sufficient data to train on and covering a reasonably large protein interaction list. So, unless explicitly indicated otherwise, we only report the results obtained using the inclusive 867-parameter set with the maximum-score detection rule in the remainder of this manuscript.

During our discussion of the PPI network in yeast, we use and refer to two training and testing PPI datasets. The ***closed set ***contains the PPIs in which only the domain-domain pairs that are optimized in the DomainGA runs exist. In other words, every domain-domain pair appearing as building blocks of the proteins in the closed set PPI list is treated as an optimization parameter. In contrast, in the ***inclusive set***, PPIs that contain at least one of the optimized domain pairs are included in the training data. Thus, the inclusive training set may contain PPIs in which there exist domain pairs that are not parameters in the GA optimization. To use an analogy, the closed and inclusive sets correspond to intersection and union combinations of the data, respectively. Further details of how these datasets were selected are given in the Methods section. Also, as discussed in the Methods section, there can be domain-pairs in the closed set that do not get optimized and get assigned a random score to put them in the fuzzy category. This is because some of the domain pairs chosen for optimization appear only in combination with the neglected domain pairs in the PPI lists. Because of the way closed set is defined, these interactions are not included during the closed set determination, and therefore, these domain pairs cannot be truly optimized in the GA runs. Thus, the optimization procedure sets their scores randomly as indefinite parameters. Because of this, even though the starting number of parameters selected for inclusive set and closed set is the same, the actual number of optimized parameters is smaller in the closed set studies (Table 1). Note that our approach implicitly assumes that the domain pairs not included as optimization parameters do not contribute to determining whether two proteins interact. This is equivalent to assuming that those domain pairs have a zero interaction score; that is, they do not interact.

The ***explanation ratio ***of the training dataset can be one evaluation criteria to determine the success of a machine-learning method. The explanation ratio is defined as the percentage of the PPIs in the training set that are successfully accounted for at the end of an optimization, i.e., it is the ratio of correctly predicted to the total number of entries in the list. Let TP, TN, FP, and FN respectively stand for true- and false-positive and negative predictions. Then, the explanation ratios of the training sets are TP/(TP + FN) and TN/(TN + FP) for the positive and negative PPI lists, respectively.

#### Optimization evaluation – parameter space search

One major concern in a parameter optimization study is the appropriate sampling of the parameter space. In the GA runs, initial values of the parameters were picked randomly and the optimized parameter values were statistically analyzed. Results reported in Figures 2 &3 are representative of our typical findings. In these figures, each row shows the optimized values of the parameter set in a particular GA run, and a uniform shade across a column means that particular parameter has the same optimized value at the end of every GA run. It is clear that optimal solutions of the GA runs have insignificant variations in the optimized parameters when the score of a parameter is in the low or high categories; that is, if the parameter indicates that a domain-domain pair is found to be interacting or not. Domain-domain interaction parameters that are in the fuzzy range, i.e., may or may not interact, generally have larger variations. This is to be expected because these fuzzy parameters are indefinite and do not contribute much to the information content of the machine-learning step. Thus, the overall explanation ratios of the training set are rather insensitive to their variations.

#### Optimization evaluation – cross validation

One way to evaluate the performance of the optimizations is the cross-validation with a testing dataset. *N*-fold cross validation with *N *~10 is typical in machine learning studies where ~10% of the entries are used for testing the predictions based on training with the remaining 90%. This process is repeated 10 times for the data split 10 ways. Performed 10-fold cross validation test using the inclusive 867 parameter dataset indicated that the DomainGA optimization achieves an average explanation ratio of 94.8% and 97.0% for the training and 92.9% and 97.0% for the testing sets for the positive and negative PPIs, respectively. We have also performed a two-fold test where half of the dataset was used as the training data, while the remainder served as the testing dataset. In this most severe form of *N*-fold cross validation, DomainGA optimization achieves an average explanation ratio of 95.7% and 96.6% for the training and 88.8% and 96.5% for the testing sets for the positive and negative PPIs, respectively. Overall, these are very respectable results for *N*-fold cross validation tests.

#### Optimization evaluation – benchmark measures

We note that the information contained in the calculated explanation ratios relates to the content of the Receiver Operating Characteristic (ROC) curves that are often reported in machine-learning studies [36-38]. Our DomainGA approach achieves explanation ratios that are larger than 95% for the parameter sets that we have used (Table 2). We also evaluate the performance of our DomainGA method using the sensitivity = TP/(TP + FN) and specificity = TN/(TN + FP) definitions given by Lin et al. [36]. Note that these properties are equal to the explanation ratios for the positive and negative PPI lists, respectively, that are reported in Table 2. Thus, our optimizations typically result in predictions with a > 95% sensitivity and > 95% specificity, which is equivalent to a point (0.05, 0.95) in the ROC plot indicating a very steep curve, a highly desired attribute.

Martin et al. developed their own set of definitions for performance evaluation [22]. They define the additional benchmark measures of accuracy = (TP + TN)/(TP + FP + TN + FN) and precision = TP/(TP + FP). Obtained values for these measures are reported in Table 2. Accuracy and precision of the DomainGA predictions with the inclusive 867 parameter set are 2.7 and 17.7 times higher than the random predictions, respectively. Having a much better precision with the closed set compared to the inclusive set is most likely due to the implicit assumption that the excluded parameters do not contribute to the predictions. This assumption is not needed in the closed set studies but it can be severe for certain protein pairs and may limit the precision of the predictions in the inclusive set cases. Therefore, as the representation is contained in itself, even though the number of truly optimized parameters is less (Table 1), optimization with the closed datasets can achieve a much higher precision. Another trend that is obvious in our results is that the precision decreases with the increase in the number of included parameters. As discussed above, this is most likely due to the limitation with the amount of information to reliably optimize some of the parameters included in the larger 2466 and 5095 parameter sets.

We have compared the predictions of our DomainGA method to the results obtained by the Maximum Likelihood Estimation (MLE) method of Deng et al. [29]. During our implementation of the MLE method, we have experimented with various false positive and negative prediction rates, which are necessary parameters during the likelihood maximization stage. We have noticed that the overall results are rather insensitive to the used false negative and positive prediction rates. The same conclusion was also reached by Deng et al. themselves [29]. Results for the MLE prediction are reported in *Supplementary Table 1 *[see Additional file 1] for various case scenarios. Explanation ratios (which also correspond to the sensitivity and the specificity) achieved by the MLE method are slightly lower than our DomainGA predictions. The accuracy obtained by the MLE is 90% for the closed and 94% for the inclusive datasets, which are lower than the accuracy of the DomainGA results, 96% and 99%. However, the most notable difference is in the precision of the predictions. Even though the precision of the DomainGA may appear to be low, 91% for the closed and 39% for the inclusive sets, it is considerable higher than the precision of the MLE method, 55% for the closed and 30% for the inclusive sets. It should be noted that both methods perform much better than the random predictions.

#### Optimization evaluation – cross verification

Although cross validation in machine learning studies is important, when the training and testing data are of the same origin, this may bias the predictive power of a method. For this reason, we have also computed the cross-explanation ratios for the DomainGA optimization results (Tables 3 &4), which help us to verify our results across datasets of different origin. In the cross verification tests, we optimize the parameter set using one set of training data (MIPS yeast data in this case) and then check the predictive power of the optimized parameter set by computing the explanation ratio of another set (e.g., another yeast dataset or the human PPI data) that has not been used during the training. We note that this is analogous to an extreme form of cross-validation because training and testing sets may not have much resemblance; therefore, an algorithm passing this type of testing would show its wider predictive power and applicability. This argument is also valid for using the closed and inclusive set combination from the same resource for training and testing purposes, albeit to a lesser degree.

**Table 3:** Cross verification with the yeast datasets

**Training set**	**Test set**		**Explanation Ratio (%) **^a^
867 pmt	344 pmt	Positive	99.3
		
inclusive	closed	Negative	95.4

344 pmt	867 pmt	Positive	69.9
		
closed	inclusive	Negative	64.3

2466 pmt	1216 pmt	Positive	98.6
		
inclusive	closed	Negative	96.0

1216 pmt	2466 pmt	Positive	76.7
		
closed	inclusive	Negative	66.5

5095 pmt	3060 pmt	Positive	99.3
		
inclusive	closed	Negative	95.6

3060 pmt closed	5095 pmt inclusive	Positive	84.5
		
		Negative	67.8

867 pmt	Uetz et al. ^b^	Core	78
		
inclusive		Full	75

344 pmt	Uetz et al. ^b^	Core	78
		
closed		Full	75

^a ^Explanation ratios were calculated by using the indicated yeast datasets as the testing data after the domain-domain interaction score parameters were optimized using the reported MIPS set as the training data in the DomainGA runs.

^b ^Explanation ratios were calculated by using the indicated closed Uetz et al. yeast datasets [1] as testing data. Uetz et al. datasets  contain only positive PPIs so test statistics were computed only for the positive predictions. *Yeast Core *set originally contained 5952 positive PPIs of which 74 were retained after selecting the entries according to their relevance to the parameter set utilized in optimizations. In the *Yeast Full *dataset case the corresponding total and retained values were 17471 and 119.

**Table 4:** Cross verification with the human PPI*

**MIPS Training set**	**Closed HPRD Test set**	**Explanation Ratio (%)**
344 pmt	Positive	75.4
	
closed	Negative	92.9

867 pmt	Positive	75.5
	
inclusive	Negative	93.7

Random	Positive	70.0
	
scores	Negative	35.9

* Explanation ratios were calculated by using the indicated closed human PPI [5] datasets as testing data after the domain-domain interaction score parameters were optimized using the reported MIPS set as the training data in the DomainGA runs. Explanation ratio is simply the ratio of successful predictions to the overall number of entries in the used positive or negative PPI list. Human PPI dataset was obtained from the Human Protein Reference Database (HPRD) and it has contained 364645 positive and ~40 million negative PPIs. Of these 215 and 6892 were retained respectively after selecting the entries according to their relevance to the parameter set utilized in optimizations.

**Figure 5:**
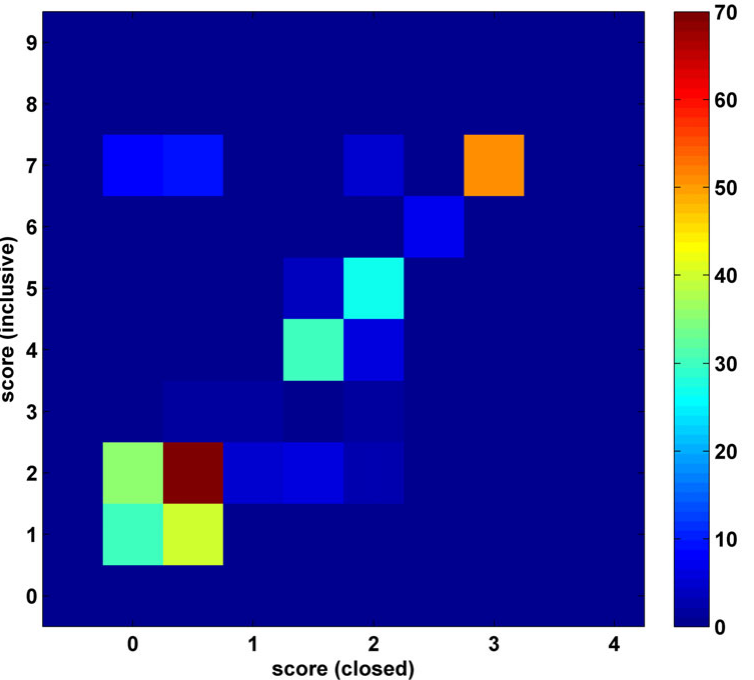
Score comparison of the 344 parameters that are common in the closed 344 parameter (x-axis) and inclusive 867 parameter (y-axis) datasets. The maximum score detection rule was used and the reported scores are the averages of the GA runs after the infrequently occurring parameter values are discarded during analysis. Each (*x,y*) entry in this histogram plot reports the number of parameters that has mean values of *x *and *y *when the referred closed and inclusive dataset was used in the optimization, respectively.

Analysis of the yeast results shows that when MIPS datasets are used for training, DomainGA optimization can achieve remarkable explanation ratios of the training datasets, typically at higher than 95% level (Table 2). Since all of the domain pairs that appear in the used training set are included as parameters in the optimization, as expected, explanation ratios are slightly higher for the closed set cases. Using the optimized parameter values, we have computed the cross-explanation percentages between the MIPS yeast datasets. These calculations (Table 3) showed that parameters optimized using the inclusive set explains the closed set data extremely well – typically at the 99% and 96% level for the positive and negative PPIs, respectively. These ratios are nearly as good as the ratios obtained by training on the closed set itself (Table 2). This may be expected because, as they are a subset of the inclusive set, the closed set data are included in the computations. On the contrary, the parameters optimized using the much more limited closed set are less successful in explaining the inclusive datasets (Table 3); however, its success is still quite respectable. Since the closed set starts to represent the inclusive set better, the cross explanation ratios improve with the increase in the size of the parameter set, Table 3. As a further check, comparison of the optimized parameter scores shows that the use of the closed and inclusive datasets results in very similar parameter values (Figure 5). We note that the parameters whose optimized values disagree between the methods appear as off-diagonal elements in the lower right or upper left corners in Figure 5; clearly, only a very small percentage of the parameters exhibit this behaviour.

Not surprisingly, cross-verification studies between the MIPS and Uetz et al. yeast datasets resulted in lower explanation ratios (Table 3). However, we observe that the explanation ratios are still at a very respectable ~75% level. Information about the PPI networks in yeast collected in various high-throughput studies is known to have small overlap [10]. This is expected to be reflected in our scheme as well where the domain-domain pairs that we have selected to represent the MIPS datasets may not contain the necessary decisive information that represent the Uetz et al. datasets [1,39]. Corollary to this would be that the domain-pairs that are important to represent the Uetz et al. data were not included in our optimization studies because, based on their occurrence, they were not among the most important ones in representing the MIPS PPI dataset.

In another cross-verification study, we have used the domain-domain interaction scores that were optimized using the MIPS yeast data and computed the explanation ratios for the human interactome (Table 4). Explanation ratios obtained for the closed sets were 74% and 93% for the positive and negative PPI sets, respectively. These are surprisingly high percentages, particularly for the negative protein interaction predictions. Explanation ratios obtained with the DomainGA method can also be compared to the predictions of a random score scheme. As Table 4 indicates, our DomainGA method significantly improves on the random predictions, particularly for predicting the non-interacting protein pairs. Accuracy (93%) and precision (28%) of the DomainGA is much higher than the corresponding values for the random predictions with 37% accuracy and 3.3% precision. Thus, the DomainGA increases the precision of the across-organism predictions by a factor of 8.4 and, based on our severe cross-verification test, we conclude that our DomainGA method shows great promise to be applicable across multiple organisms.

### D. Evaluation of the obtained domain-domain interaction scores

**Figure 6:**
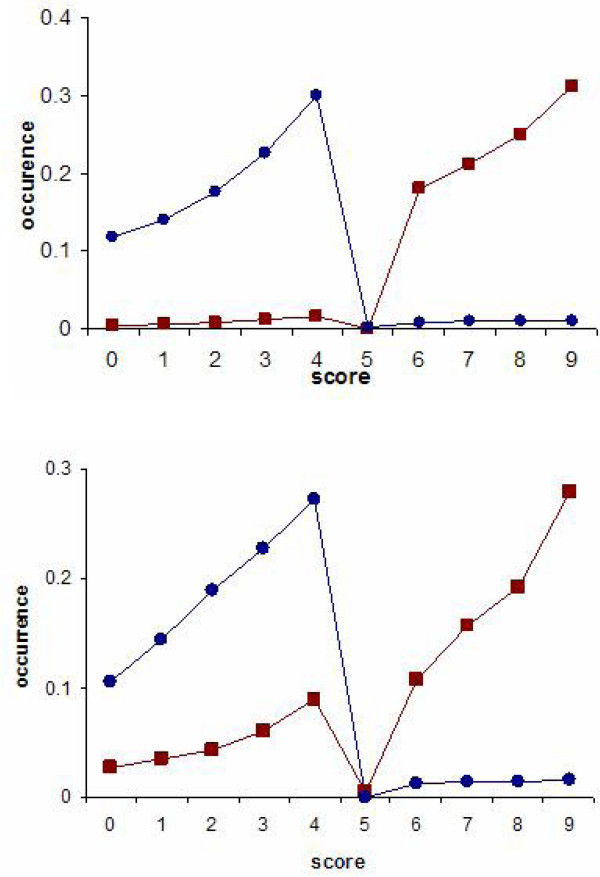
Comparison of the strengths of the MIPS positive (red line with squares) and negative (blue line with circles) protein-protein interactions computed using the DomainGA optimized domain-domain interaction scores. Vertical axis shows the percentage of the PPIs with interaction scores that were calculated by binning the total protein-protein interaction scores using unit bin sizes. Top: Inclusive set yeast PPI; Bottom: Closed set human PPI.

As discussed in Part A above, a rationale behind the presented research was the lack of discriminatory power of the InterDom domain-domain interaction scores. To further evaluate the DomainGA method's performance, we have performed a similar analysis using our interaction scores. Figure 6 reports the distributions of the predicted yeast PPI scores obtained using the domain-domain interaction scores obtained in the inclusive 867 parameter study. Using the same optimized parameter values, as in the cross-verification study reported above, Figure 6 also reports the predicted score distribution for the human interactome for the closed PPI dataset. For both cases, distributions for the positive and negative PPI scores are clearly well separated indicating that, in terms of having discriminatory power, our DomainGA method significantly improves on the InterDom scores.

The *Supplementary Table 2 *[see Additional file 1] presents the full set of our optimization results obtained using the inclusive 867 parameter MIPS data and its corresponding 344 parameter closed dataset as the training data in the DomainGA approach. The same table also tabulates the histograms for the scores of individual domain-domain pairs and compares the maximally observed score with the mean values of the GA runs. The results of the two optimization studies agree very well, which is also evident in Figure 5.

### E. Optimization using only the positive PPIs

Although the positive PPI lists are generally based on direct experimental observation, the negative protein-protein interactions can be ambiguous. As in the compilation of the MIPS dataset that we have used, negative interactions are often extracted by making certain assumptions; for example, proteins that occupy different sub-cellular compartments do not interact. Implicit in this assumption is that the proteins would still not interact even if the biophysical barriers keeping them in separate compartments are removed. This in essence is a severe assumption whose correctness is questionable, and the assignment of locations can itself be problematic [40]. To test the utility of DomainGA without any negative PPI dataset, we have experimented with a different GA optimization fitness function that maximizes the explanation ratio of the training dataset while keeping the values of the domain-domain score parameters at a minimum. The idea of minimizing the number of positive domain interactions is analogous to choosing a smaller set of domain-pairs with higher-specificity concept that was advocated in Ref. [33].

**Figure 7:**
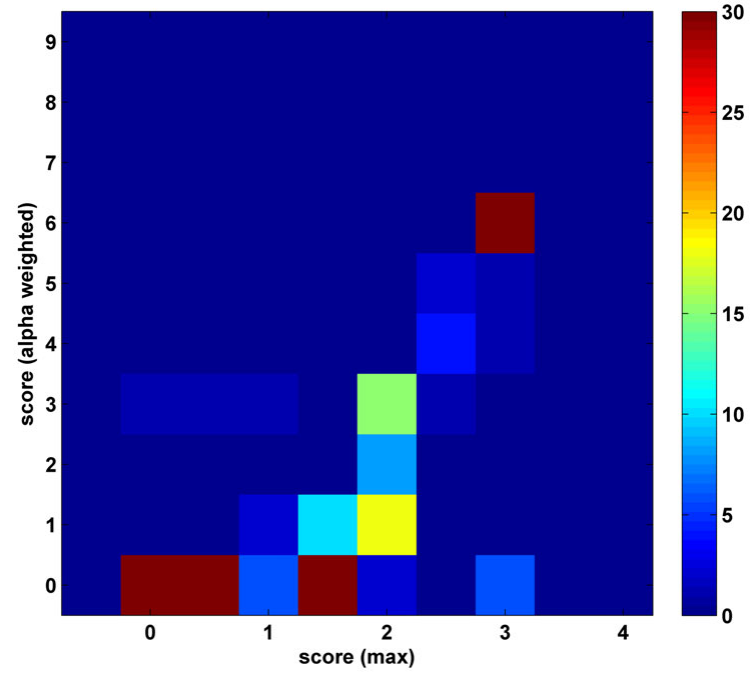
Comparison of the mean scores of the parameters that were optimized using the 344 parameter closed set training data with different fitness functions. X-axis: Optimization using both the negative and positive PPIs with the maximum score detection rule (as in Figure 4). Y-axis: Optimization with the minimum parameter magnitude fitness function using only the positive PPI list. The maximum value of the color scale is lowered from 121 to 30 to enhance the contrast between the histogram points.

Comparison of the results obtained using only the positive MIPS PPI dataset for the closed 344 parameter case with the new minimum parameter magnitude fitness function (details of the optimization routine are described in the Methods section) with the above reported results shows very good correlation between the results (Figure 7). In line with the earlier cases, the explanation ratio of the training set was very high (98%). To test whether the unused negative PPI list was still well predicted with the obtained scores, we computed its explanation ratio, and it was 96%, an excellent ratio. Thus, we can confidently state that with the use of realistic fitness functions in the GA optimization runs, one may be able to sidestep the problems associated with the availability of the negative PPI training data.

One clear trend in the optimized values of the parameters is that scores for the domain-domain parameters are generally lower with the new optimization fitness function (Figure 7). This is an expected outcome because, as a result of the way the optimization fitness score is constructed, the algorithm would only set a minimal number of parameters to have large non-zero values. We note that the shift in the values of the scores does not create any discrepancy between the results. There are 44 parameters that have values > 5 in the optimization with the new fitness function. This finding for the number of interacting domain pairs is in accord with the predictions of the closed set optimization runs that use both the positive and negative PPI lists as training data [see the detailed scores in the Supplementary Table 2 in Additional file 1].

### F. Testing against structurally identified interactions

The iPfam resource [41] makes use of the biomolecular structures deposited in the protein data bank (PDB) and identifies the possible interactions between the domains defined by the Pfam classification. Because it is based on structural information, derived domain-domain interactions can be considered reliable. However, it should be kept in mind that iPfam uses an automated computational approach and does not distinguish between biological and crystal contacts. In addition, interactions between the domains of a single polypeptide and domain interactions between separate peptides are not treated separately. These characteristics can lead to false-positive detections in iPfam.

We have downloaded the list of domain interactions between Pfam-A class domains from the iPfam website and investigated the corresponding scores that were obtained in our DomainGA studies. Out of the 867 domain-domain pairs included in our most precise optimization study, 33 are included in the iPfam list (Table 5). According to our score predictions with the inclusive set, seven of these domain pairs have low scores thus reflecting a disagreement between our results and the information listed at the iPfam database. In addition, the score for one domain pair, WD40-Ggamma, has discrepancy between the values obtained using the inclusive and the closed datasets. Detailed investigation of these eight conflicting cases can be illuminating for evaluating the success of the DomainGA method.

**Table 5:** Domain-domain interaction scores for the pairs that appear in the iPfam database*

**Domain Name (Pfam ID)**	**Domain Name (Pfam ID)**	**Mean Score ^a ^(Inclusive/Closed set)**
PNPase (PF03726)	RNase_PH (PF01138)	7.61/7.48
GTP_EFTU (PF00009)	GTP_EFTU (PF00009)	7.57
Ribosomal_L6 (PF00347)	Ribosomal_L6 (PF00347)	7.56
CK_II_beta (PF01214)	CK_II_beta (PF01214)	7.56/7.49
Prenyltrans (PF00432)	PPTA (PF01239)	7.53
Ribosomal_S8 (PF00410)	Ribosomal_S2 (PF00318)	7.52
TPR_1 (PF00515)	TPR_1 (PF00515)	7.52
Ribosomal_S11 (PF00411)	Ribosomal_S7e (PF01251)	7.52/7.50
IF-2B (PF01008)	IF-2B (PF01008)	7.51/7.49
CK_II_beta (PF01214)	Pkinase (PF00069)	7.49
Ribosomal_S2 (PF00318)	Ribosomal_S2 (PF00318)	7.49
Bromodomain (PF00439)	Bromodomain (PF00439)	7.48/5.66
WD40 (PF00400)	G-gamma (PF00631)	7.48/1.97*
Ribosomal_L4 (PF00573)	Ribosomal_L37e (PF01907)	7.48
G-alpha (PF00503)	WD40 (PF00400)	7.46
PFK (PF00365)	PFK (PF00365)	7.45
Ribosomal_S8e (PF01201)	Ribosomal_S2 (PF00318)	7.44
GTP_EFTU (PF00009)	EF1_GNE (PF00736)	7.43
Proteasome (PF00227)	Proteasome (PF00227)	6.26/6.11
ATP-synt_ab (PF00006)	ATP-synt_C (PF00137)	5.86
Clat_adaptor_s (PF01217)	Adaptin_N (PF01602)	5.40
Ribosomal_L4 (PF00573)	Ribosomal_L15e (PF00827)	5.38
Glyco_transf_20 (PF00982)	Glyco_transf_20 (PF00982)	5.33
Ribosomal_L24e (PF01246)	Ribosomal_L14e (PF01929)	5.30/5.39
Prefoldin (PF02996)	KE2 (PF01920)	4.91
Proteasome (PF00227)	AAA (PF00004)	4.75/4.92
Pkinase (PF00069)	Pkinase (PF00069)	2.43*
WD40 (PF00400)	WD40 (PF00400)	2.11/2.04*
RRM_1 (PF00076)	RRM_1 (PF00076)	2.07*
Pkinase (PF00069)	Ank (PF00023)	2.05*
Myb_DNA_binding (PF00249)	Myb_DNA_binding (PF00249)	2.04*
WD40 (PF00400)	PH (PF00169)	1.92/2.04*
Ank (PF00023)	Ank (PF00023)	1.87*

* Entries are discussed in the main text.

^a ^Scores obtained in the inclusive 867 parameter study. In the entries that contain more than one score, the second numbers are the scores for the domain pairs that were obtained in the corresponding 344 parameter closed set optimizations.

Two of the domain pairs, WD40-PH and WD40-WD40, have low scores in optimizations using both the closed and the inclusive training data. Thus, these two domain-domain pairs are consistently detected to be non-interacting with our method. As discussed in the iPfam website, WD40 repeats are short amino acid motifs, and proteins that contain WD40 have a large number of repeating units of this domain. Repeating units generally form a beta-propeller structure, which is believed to serve as a scaffold for protein interactions. When we investigated the structural evidence presented at the iPfam website for the WD40-WD40 domain pair interaction, all of the observed interactions are between the WD40 domains of the same polypeptide chain (i.e., they are intra-peptide interactions) and are mainly between the WD40 domains of the same beta-barrel. We note that our method detects the PPIs between two distinct proteins so the interactions between the pairing domains should be between different polypeptides (i.e., inter-peptide interaction). Therefore, we believe that the low score obtained by DomainGA for the WD40-WD40 interaction is reasonable. This finding shows the importance of differentiating the interactions between two or more peptides and between the domains of the same polypeptide [42].

The pleckstrin homology (PH) domain consists of about 100 residues and is involved in a wide range of intracellular signaling processes. The 3D structure of PH domain contains two perpendicular anti-parallel beta sheets and an amphipathic helix. There is only one PDB file in which the interaction between WD40 and PH domains is observed, and it is unclear how specific that observation is. Therefore, the low score obtained with the DomainGA method is very likely correct. We would like to emphasize that, even if our prediction is wrong, detection of such discrepancies between methods like ours and the direct structural observation is important because it helps to focus future studies to answer specific questions.

In the WD40-Ggamma case, DomainGA prediction with the inclusive training dataset is positive, and the prediction with the closed dataset is negative. G-gamma domain is found in the gamma subunit of the heterotrimeric G protein complexes and in regulators of G-protein signaling proteins. G-gamma domain is believed to be instrumental in interactions with beta-propeller proteins. There are eight PDB structures listed at the iPfam website, and five of them report direct WD40-Ggamma domain interaction between two polypeptide chains. So there is reasonable evidence that these two domains interact, and therefore, DomainGA prediction with the inclusive training dataset seems to be more correct.

For the other five domain interaction pairs we only have the score for the optimization with the inclusive dataset (Table 5). The ankyrin repeat (Ank) motif is one of the most abundantly occurring protein domains. It is an ~33 amino acid-long module, and this tandemly repeated domain is contained in many proteins with very divergent functionalities. The iPfam website reports 21 PDB structures containing the Ank-Ank domain interaction; however, only one of those is between two separate peptide chains, and only a few structures contain intra-peptide type Ank-Ank interactions. We therefore predict that the Ank-Ank interaction is most likely negative.

PKinase domain represents the catalytic core common in both the serine/threonine and tyrosine protein kinases. Activity of some of the kinases involves a biomolecular dimerization step, which may be facilitated by the PKinase domains of the proteins. Therefore, prediction of the DomainGA for this particular domain pair is most likely incorrect.

Although the iPfam site lists four PDB structures with the PKinase-Ank interaction, these may simply be caused by crystal packing. The Ank domain is mainly defined by its structure rather than its function; that is, it has no direct functional relevance to the kinase activity. Therefore, whether it would interact with the catalytic core of the kinases is highly questionable. Our analysis raises questions about the interaction between these two domains. In this case, more detailed investigation is needed, which is one of the intended purposes behind the development of the DomainGA method.

The remaining two domain interactions are for the DNA- and RNA-binding proteins. The Myb_DNA-binding domain is found in Myb proteins where three tandem repeats have been shown to be involved in DNA-binding. The iPfam site lists four PDB structures where the interactions between two Myb_DNA-binding domains are observed only between the two domains of the same polypeptide; that is, of intra-peptide-interaction origin. Therefore, the evidence for this domain-domain interaction in causing a PPI is evasive, and the results from DomainGA may be indicative of this.

The RNA recognition motif RRM is characteristic of an RNA-binding protein and is found in a variety of RNA-binding proteins. This interaction has been observed in 26 PDB files where it was detected between separate peptides. Thus, this interaction may facilitate the formation of a complex between multiple strands, and it may be real.

As mentioned earlier, we stress that regardless of their correctness, observed differences between our findings and the information available at other sources actually make our method more appealing. In addition to helping with constructing PPI networks, our domain-based approach may also be of use in detecting the biophysical properties of the protein functional domains.

## Conclusion

Because the high-throughput experimental methods to identify PPIs can be expensive and inaccurate, computational methods can nicely complement experimental approaches and validate experimental observations. The usefulness of machine-learning techniques such as Support Vector Machines (SVMs), Artificial Neural Networks (ANNs), and Bayesian Networks has been demonstrated in several biology problems, such as homology detections and protein-protein interaction prediction [12,22,38,43-45]. Classification of domain-domain interactions can be possible through an analogous use of SVM or ANN, which, for a given kernel and network structure, respectively, are deterministic approaches. However, for the problem addressed in this study, the large number of parameters (domain-domain interactions) and the sparseness of the training data may make the deterministic method too dependent on the used kernel or the network structure. For this reason the use of probabilistic search methods such as the GAs can be a more advantageous optimization approach for the types of studies reported here.

In this study, we have developed the DomainGA method, which predicts the protein-protein interactions using the protein functional domain information and tested its usefulness on the model organism *S. cerevisiae*. Because of the limitation imposed by the amount of available training data, in its current version we have included only a small number of domain-domain interaction pairs as prediction parameters. As more experimental data become available, the reported scores can be improved and the domain parameter set can be expanded. Our attempts with the larger 2466 and 5095 parameters show that this is possible when there is enough training data and that it is feasible to handle the added computational complexity. In addition to dealing with the PPI data specific to a specific organism, we are in the process of combining the data from multiple organisms to create larger training and testing datasets. Based on the encouraging results obtained in our cross-verification tests where scores optimized using the yeast data were used to predict the human PPIs, we expect that combining the data from multiple organisms will increase the predictive power of our approach.

It should be noted that it may be possible to extract the domain interaction information from the PPI lists using the domain co-occurrence statistics [27,28]. To compare the predictions of our DomainGA with the domain occurrence statistics, we have computed the pair likelihood ratios using a very simple approach (Methods section). Although the results show reasonable agreement, the R-square value for the overall correlation is rather low, r^2 ^= 0.71. In addition to using plain co-occurrence ratios, expectation maximization-based approaches can be used to derive the likelihoods associated with the domain co-occurrences, and these represent the probabilities of interaction [28,29,32]. As reported in the Results C section, in addition to the improvement in the accuracy, precision of the DomainGA algorithm predictions is considerably better than that of the maximum likelihood estimation method [29].

While extracting the protein domains, it has been implicitly assumed that the variations in the amino acid composition of the same domain type among proteins do not alter the domain's interaction patterns. As amino acid substitutions may impact complex formation affinities, disregarding the exact sequence of the functional domains may lead to failures in some cases. Inclusion of such local structural characteristics can be very useful in predicting the effects of mutations [46] and alternate splicing events [47]. They can have implications in biomarker and pharmaceutical research by helping with target identification [48,49], and they can be used to facilitate better bait selection prior to high-throughput experimentation. Even though the necessary computational extension to include the local amino acid sequence dependence is straightforward, inclusion of the amino acid composition of the functional domains into the interaction score scheme would require a combinatorial increase in the needed training dataset sizes. Such generalizations are currently impractical, but they will be included in future studies as such details are warranted.

Recent developments in high-throughput protein chip technologies are good indicators of the DomainGA method's potential. For example, Jones et al. recently investigated the interactions between various domains of the proteins involved in cell-signalling cascades [50]. Measured binding affinities are directly related to the domain-domain interaction scores developed in this study. As they become available, such complimentary experimental studies can be used to benchmark the predictions of the computational methods.

The possibility of false predictions is unavoidable in any computational method [2,12,22]. This may limit the usefulness of the computational protein-protein interaction predictions to supplement the experimental observations. Keeping this in mind, we envision the DomainGA as a first step of a multi-tier approach to constructing PPIs. As it is based on fundamental structural information, the DomainGA approach can be used to create the potential PPIs, and the accuracy of the constructed interaction template can be improved later using complementary methods such as those based on literature search or other prediction methods. Obtained explanation ratios during the reported test case studies clearly show that the false prediction rates of the obtained templates would be reasonably low and can be lowered even further with additional secondary tests.

## Methods

Genetic Algorithms (GA) can be used as a search technique to find best-estimate solutions in optimization problems. They are a particular class of machine-learning algorithms that uses techniques inspired by evolutionary biology such as inheritance, mutation, natural selection, and recombination. GAs are typically implemented as a computer simulation in which a population of abstract representations (called chromosomes) of candidate solutions (called individuals) evolves toward better solutions. The solutions are either strings of 0/1s or can have different encodings. The evolution starts from a random population, and changes occur through a selection process over generations. In each generation, the fitness of the whole population is evaluated, and most successful individuals are kept for the next generation. This selected group of individuals is supplemented with offspring that are obtained by modifying (random mutations and/or recombinations obtained using crossovers with inherited characteristics) the individuals that are stochastically selected from the current population (with probabilities based on their fitness). The set formed this way then becomes the current population set in the next iteration of the algorithm.

Each chromosome in the DomainGA is an array of domain-domain interactions (parameter set to be optimized). We start with 50 chromosomes that have randomly initialized parameter values as their array elements. In this study we use an integer scale of [0-T] where T was 9 in all cases except in initial studies testing the dependence on the scale. In each generation the population size is increased 10-fold by the use of recombination, mutation, and random-generation operations. A multi-point recombination function is used among randomly selected chromosomes to add 250 (5×) more chromosomes. Random mutations are carried out on the genes of the chromosome (parameters) to create 150 (3×) new chromosomes. Finally, 50 (1×) random chromosomes are created and added to the initial population. When combined, this set forms the population of a particular generation. In the later iteration stages of the GA run, the 50 seed chromosomes are selected by ranking the population according to the optimization fitness function and selecting the top 50 entries.

### A. Optimization fitness function

Our GA runs used an optimization fitness function that describes how well the training PPI set is explained by the chromosome population. Each chromosome is an array of scores for the included domain-domain pairs, and this score set can be used to decide whether two proteins interact. Adapting the domain-domain interaction scores to predict PPIs requires the development of a criterion to decide which type of domain-domain score corresponds to a PPI. For this, we first form a list of all possible domain-domain interactions between two proteins; that is, all possible combinations between domain pairs. We then take either the largest (maximum score detection rule) or the sum (total score detection rule) of the domain-domain interaction scores from this list to represent the strength of the interaction between the proteins. If the determined strength is larger than a pre-assigned cutoff (> 5 when T = 9), then that pair of proteins is predicted to interact. The pair is assumed to not interact if the score is below the cutoff (< 5 when T = 9), and an indecisive assignment is made if it is equal to the cutoff. These PPI predictions are then compared to the training data where correct prediction is granted + 1 point, and a penalty of -1 is applied for an incorrect identification. Indecisive assignments do not contribute to the optimization fitness score. As the number of positive and negative PPI entries in the training dataset can be vastly different, we normalize the contributions of the negative and positive PPIs to the overall fitness function according to the number of PPIs in each list such that both lists carry equal weight. The optimization fitness function is then maximized during the GA iterations, and when the score does not change over 15 successive iterations, the GA is terminated. At least 2000 GA runs starting from randomly selected populations were executed for each reported case. Results from the GA runs whose converged fitness function scores are low are considered unsuccessful, and those runs are discarded from the statistical analysis that determines the distribution and mean values of the optimized parameter values [e.g., Supplementary Table 2, in Additional file 1].

In the GA runs that use only the positive PPI list as the training data, we have also used a fitness function that minimizes the magnitude of the involved parameters. This fitness function has two terms. The first term represents the explanation ratio of the training dataset; it is exactly the same function that was discussed in the previous paragraph. The second term is the sum of the magnitude squares of the parameters; that is, the sum of squares of all domain-domain interaction scores. We multiply the second term with a weight factor α and then subtract it from the first term and use the resulting function as the fitness function in the GA runs. Results reported in Figure 7 were obtained using α = 0.5, and the maximum score detection rule was used in deciding the PPI predictions. This fitness function maximizes the explanation ratio while assigning a minimum number of domain pairs as interacting partners. Note that without the second term, assignment of high values to all the optimized parameters would lead to a perfect explanation ratio of the positive PPI list so it would be a trivial global solution. The subtracted weighted parameter magnitude term blocks the optimizer from assigning high parameter values unless they are necessary to achieve a good explanation ratio.

### B. Data selection

Success of a learning algorithm-based method depends on the quality of the available training data. For the PPI network construction studies, the training dataset ideally contains a list of truly positive interactions (i.e., real PPIs) as well as a list of non-interacting pairs of proteins (i.e., true negatives). A well-performing interaction scoring scheme should have predictive power and be able to discriminate between the true and false observations. However, as briefly mentioned in the Background section, definite identification of true and false PPIs is problematic. Therefore, one has no choice but to create positive and negative PPI lists and assume that the list is correct.

Fortunately, there are efforts devoted to construct PPI lists for yeast that are as reliable and correct as possible [12,14,22]. We obtain our training and test datasets from these earlier compilations. For the positive and negative yeast PPIs, we mostly use the information available at the Munich Information Center for Protein Sequences (MIPS) [35,51] and use the version originally compiled by Jansen et al. [12], which contains 8250 positive and ~2.7 million negative PPIs. This set of PPIs represents the interactions between proteins that are present in the same complex. Negative PPIs were obtained by using the protein location information by assuming that proteins residing in different subcellular compartments do not interact. Although this assumption is not entirely valid, because of the low error rate, its effect on the outcome of the prediction algorithms is believed to be unimportant.

Cross-verification tests for DomainGA scores were performed using the Core and Full yeast PPI datasets from Uetz et al. [39]. The Core subset of DIP contains the pairs of interacting proteins (ScereCR20060402 list downloaded on 04/02/2006) identified in the budding yeast that were validated according to the criteria described in Deane at al. [10]. The Full Yeast set corresponds to the subset of DIP that contains all the pairs of interacting proteins identified in the budding yeast (yeast20060402.lst file downloaded on 04/02/2006). These sets contain 5952 and 17471 positive protein-protein interactions, respectively.

The datasets originally compiled by Rhodes et al. [5] were used in the across-organism cross-verification test with the human protein-protein interactions. These sets were obtained from the Human Protein Reference Database (HPRD), and the lists contain 364,645 positive and ~40 million negative protein-protein interactions.

These PPI datasets were further processed to obtain the relevant subsets (Table 1) for use in the GA optimization runs. As shown in Tables 1, 2, 3, 4, we have formed the training and testing datasets in two different ways. The *closed set *is a subset of the PPIs such that included PPIs only contain the domain-domain pairs whose interaction scores are optimized in the runs. All the other PPIs are not selected. In contrast, the PPIs in which the involved protein pair has the potential to interact through one or more of the optimized domain-domain pairs are included in the *inclusive set*. In other words, PPIs in the inclusive set may interact through the domain-domain pair that is chosen as an optimization parameter, but these PPIs may have other domain-domain interaction pairs that are neglected in the optimizations. Details of the resulting set sizes are reported in Table 1.

In forming the closed dataset, an additional problem arises which reduces the number of parameters that can be truly optimized with the DomainGA method. Say that a domain pair is chosen as a parameter to be optimized. If all the PPIs that include this is domain pair d_ij _contain at least another domain pair whose interaction score is not optimized (i.e., not a domain pair selected as a parameter), then these PPIs will be excluded from the list defining the closed set. This would lead to the case that domain pair d_ij _may not appear in any of the PPIs defining the closed dataset. When that happens, as there is no information that is relevant for its optimization, the value of this parameter will be set randomly during the optimization. This was observed in our simulations and, when T was 9, such parameters had average values in the [4,5] range as expected. Thus, as they should, these parameters appear as fuzzy, uncertain parameters in the results. Whenever the closed and the inclusive set results are compared, these non-optimized parameters are omitted from the figures.

We derive the domain information from the InterPro database [52,53]. InterPro capitalizes on the individual strengths of a number of databases including PROSITE [54], Pfam [24], PANTHER [55], and PRINTS [56] as well as sequence-cluster based methods such as PSI-BLAST [57] on well-characterized proteins to derive protein domains. As it unifies Pfam with other databases, use of the InterPro database allowed us to obtain a better coverage for the domains of the proteins of interest.

### C. Optimized parameter set selection

As in any multi-parameter optimization approach, the involved parameter set needs to be defined in our DomainGA approach. For N domains there are N(N + 1)/2 possible domain-domain pairs whose values need to be known. Noting that N is on the order of 10^4 ^in InterPro classification, there are ~10^8 ^possible interacting domain-domain pairs. Reliable optimization of such large parameter sets requires PPI training data that are not currently available and possibly will not be available in the near future either. Therefore, inclusion of all possible domain-domain pairs in the optimization process is not realistic. To avoid parameter over-fitting, we have initially started with a very small parameter set and assumed that it is large enough to represent the important domain, and thus protein, interactions. To select the used domain-domain interaction parameter set, we have computed the histogram diagrams for the number of occurrences of the domain-domain pairs in the training PPI sets and sorted the pairs according to their occurrence counts to achieve reasonably large training and test datasets. This has allowed us to select sets with different number of domain-domain pairs (103, 867, 2466, and 5095 pairs; Table 1) to use as the parameter sets in the GA optimizations. Choice of these parameters was based on the occurrence of the domain pairs in the positive and negative standard PPI lists where roughly half of the parameters came from each list. We again stress that DomainGA implicitly assumes that the omitted domain pairs are not a determining factor in deciding whether two proteins interact. Thus this would be equivalent to assigning zero (i.e., non-interacting) values to the neglected domain pairs.

### D. Likelihood analysis based on domain occurrences

One very simple analysis method is to derive the domain interaction probabilities as the likelihoods that are computed using the relative occurrence statistics in the positive and negative PPI lists. Starting with the list of PPIs in the yeast MIPS dataset, we first obtain the domains of the proteins involved in the interactions and then create the unique list of all possible domains and domain-domain pairs amongst the protein-protein interactions. This gives the occurrence counts of each of the domains and the domain-pairs. Once the occurrence statistics is collected, the likelihood for each domain-pair in the set X (= negative or positive) was calculated by the following rule:

L(domain pair ij)=Occurrence of domain pair in the protein pair set X(Occurrence of domain i in X)(Occurrence of domain j in X)
 MathType@MTEF@5@5@+=feaafiart1ev1aaatCvAUfKttLearuWrP9MDH5MBPbIqV92AaeXatLxBI9gBaebbnrfifHhDYfgasaacH8akY=wiFfYdH8Gipec8Eeeu0xXdbba9frFj0=OqFfea0dXdd9vqai=hGuQ8kuc9pgc9s8qqaq=dirpe0xb9q8qiLsFr0=vr0=vr0dc8meaabaqaciaacaGaaeqabaqabeGadaaakeaacqWGmbatcqGGOaakcqqGKbazcqqGVbWBcqqGTbqBcqqGHbqycqqGPbqAcqqGUbGBcqqGGaaicqqGWbaCcqqGHbqycqqGPbqAcqqGYbGCcqqGGaaicqqGPbqAcqqGQbGAcqGGPaqkcqGH9aqpdaWcaaqaaiabb+eapjabbogaJjabbogaJjabbwha1jabbkhaYjabbkhaYjabbwgaLjabb6gaUjabbogaJjabbwgaLjabbccaGiabb+gaVjabbAgaMjabbccaGiabbsgaKjabb+gaVjabb2gaTjabbggaHjabbMgaPjabb6gaUjabbccaGiabbchaWjabbggaHjabbMgaPjabbkhaYjabbccaGiabbMgaPjabb6gaUjabbccaGiabbsha0jabbIgaOjabbwgaLjabbccaGiabbchaWjabbkhaYjabb+gaVjabbsha0jabbwgaLjabbMgaPjabb6gaUjabbccaGiabbchaWjabbggaHjabbMgaPjabbkhaYjabbccaGiabbohaZjabbwgaLjabbsha0jabbccaGiabbIfaybqaaiabcIcaOiabb+eapjabbogaJjabbogaJjabbwha1jabbkhaYjabbkhaYjabbwgaLjabb6gaUjabbogaJjabbwgaLjabbccaGiabb+gaVjabbAgaMjabbccaGiabbsgaKjabb+gaVjabb2gaTjabbggaHjabbMgaPjabb6gaUjabbccaGiabbMgaPjabbccaGiabbMgaPjabb6gaUjabbccaGiabbIfayjabcMcaPiabcIcaOiabb+eapjabbogaJjabbogaJjabbwha1jabbkhaYjabbkhaYjabbwgaLjabb6gaUjabbogaJjabbwgaLjabbccaGiabb+gaVjabbAgaMjabbccaGiabbsgaKjabb+gaVjabb2gaTjabbggaHjabbMgaPjabb6gaUjabbccaGiabbQgaQjabbccaGiabbMgaPjabb6gaUjabbccaGiabbIfayjabcMcaPaaaaaa@C878@

The overall likelihood was represented as the log of the positive likelihood minus the log of the negative likelihood. The scores obtained with DomainGA and this simple likelihood analysis have a correlation of r^2 ^= 0.713. Note that the calculated likelihoods can be improved using an iterative maximization approach such as the maximum likelihood estimation method that is discussed in the Results C section.

## Authors' contributions

HR conceived the idea, both authors designed the study and participated in its coordination, and MS performed the analysis. The authors wrote the paper together and approved the final manuscript.

## Supplementary Material

Additional File 1 Table 1: This sheet lists the results for the MLE prediction (Deng et al.) for various scenarios. Table 2a: This sheet lists the DomainGA parameter scores and their distributions. Table 2b: This sheet lists the names of the domains used in the DomainGA calculations. Click here for file
